# Supplementary material for: Sustainable Bio-Based Epoxy Resins with Tunable Thermal and Mechanic Properties and Superior Anti-Corrosion Performance
Source: Polymers (Basel). 2023 Oct 21;15(20):4180. doi: 10.3390/polym15204180 (PMC10610945; doi:10.3390/polym15204180)
Supplement: Supplementary file 1 [file polymers-15-04180-s001.zip › polymers-2658666-supplementary.pdf]

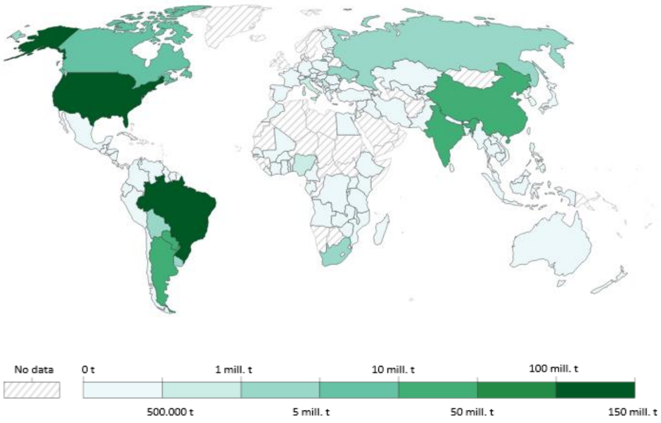

Figure S1. Public domain data of soy bean production (in tons) in 2021.

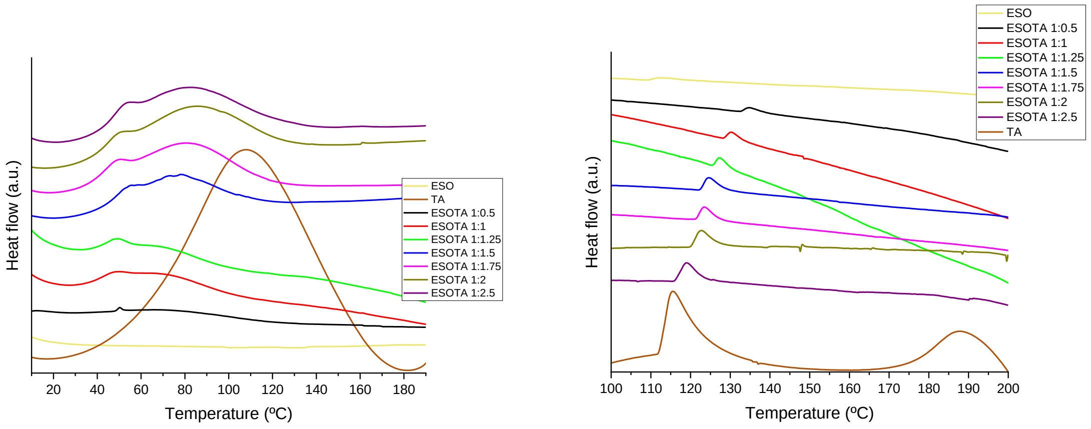

(**a**) (**b**)

Figure S2. DSC thermograms recorded during: (**a**) 1st heating scan and (**b**) 2nd heating scan following the cooling of the sample.

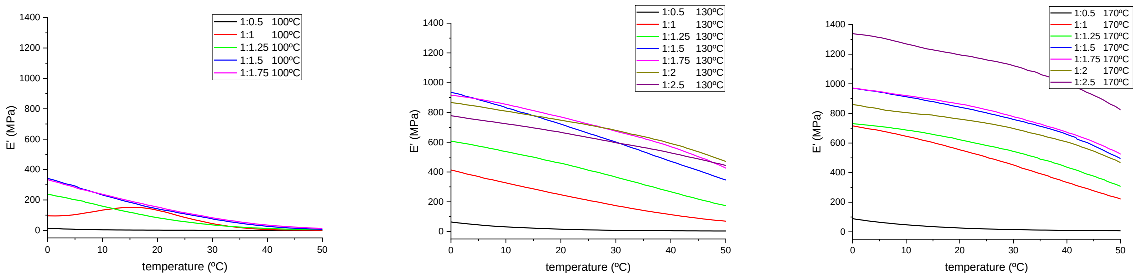

(**a**) (**b**) (**c**)

Figure S3. Magnification between 0 and 50°C of the DMTA determined storage modulus plots for each sample, cured at (**a**) 100 °C, (**b**) 130 °C and (**c**) 170 °C.

Table S1. DSC results for both initial products and cured resins.

SAMPLE	1ST TRANSITION (°C)	2ND(3RD) TRANSITION (°C)
**ESO**	-	-
**TA**	108<br>(50.1-166.6)	115.5<br>(111.9 - 123.1)<br>187.8<br>(168.1 – 200)
**ESOTA 1:0.5**	50.1<br>(36.3 - 119.0)	134.7<br>(132.6 - 140.5)
**ESOTA 1:1**	50.6<br>(35.0 - 104.6)	130.2<br>(128.3 - 134.8)
**ESOTA 1:1.25**	49.3<br>(39.1 - 99.7)	127.2<br>(125.8 - 130.5)
**ESOTA 1:1.5**	78.2<br>(38.6 - 111.4)	124.6<br>(122.4 - 129.8)
**ESOTA 1:1.75**	80.5<br>(33.7 - 119.3)	123.5<br>(121.5 - 128.2)
**ESOTA 1:2**	85.4<br>(33.9 - 124.7)	122.7<br>(120.0 - 127.5)
**ESOTA 1:2.5**	82.5<br>(36.6 - 126.4)	119.0<br>(116.1 - 123.5)
